# Supplementary material for: Cerebral Organoids: A Human Model for AAV Capsid Selection and Therapeutic Transgene Efficacy in the Brain
Source: Mol Ther Methods Clin Dev. 2020 Jun 1;18:167–75. doi: 10.1016/j.omtm.2020.05.028 (PMC7327852; doi:10.1016/j.omtm.2020.05.028)
Supplement: Document S1. Figures S1 and S2 [file mmc1.pdf]

OMTM, Volume *18*

Supplemental Information

Cerebral Organoids: A Human Model for AAV Capsid Selection and Therapeutic Transgene Efficacy in the Brain

Josse A. Depla, Marina Sogorb-Gonzalez, Lance A. Mulder, Vivi M. Heine, Pavlina Konstantinova, Sander J. van Deventer, Katja C. Wolthers, Dasja Pajkrt, Adithya Sridhar, and Melvin M. Evers

Supplemental figures

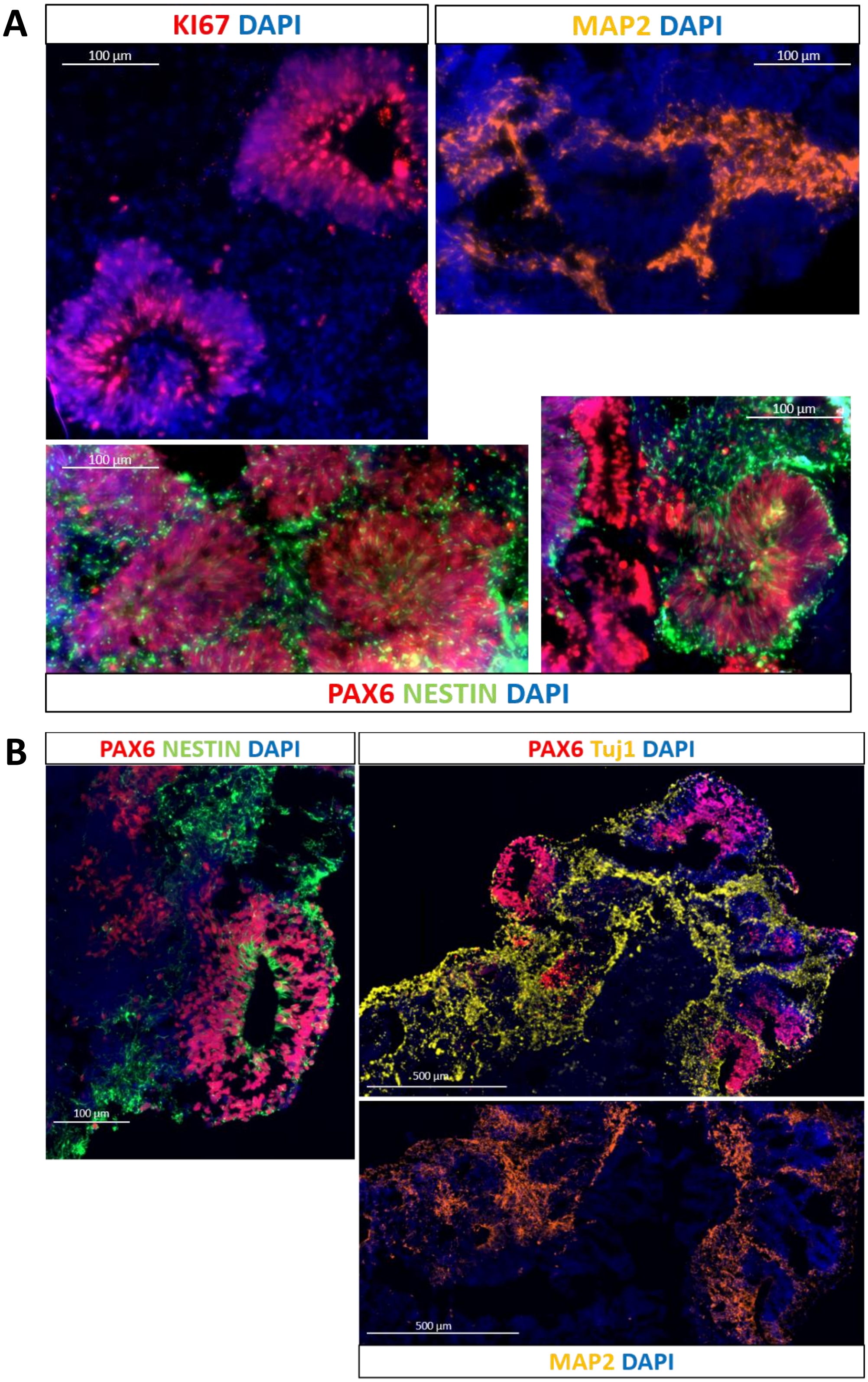

Figure S1: Generation of cerebral organoids. Images of immunostained sections of two 30 day old organoids from two different batches (A & B), show the presence of neural progenitors (PAX6), neurons (Tuj1 and MAP2) and proliferating cells (Ki67). Nuclei were dyed with DAPI.

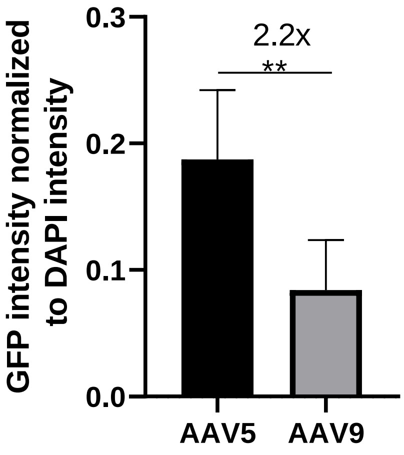

*Figure S2: Comparing rAAV5 and rAAV9 transduction efficiency in cerebral organoids. rAAV5 transduced organoids express GFP at a 2.2 times higher intensity compared to rAAV9 transduced organoids (**p<0.005). Total GFP intensity was measured in z-stacks of 12 regions per organoid. The intensity was normalized to the DAPI signal.*
