# Supplementary material for: Cerebral Organoids: A Human Model for AAV Capsid Selection and Therapeutic Transgene Efficacy in the Brain
Source: Mol Ther Methods Clin Dev. 2020 Jun 1;18:167–75. doi: 10.1016/j.omtm.2020.05.028 (PMC7327852; doi:10.1016/j.omtm.2020.05.028)
Supplement: Document S2. Article plus Supplemental Information [file mmc2.pdf]

Molecular Therapy
Methods & Clinical Development
Original Article

Cerebral Organoids: A Human Model for AAV Capsid Selection and Therapeutic Transgene Efficacy in the Brain

Josse A. Depla,[1,2] Marina Sogorb-Gonzalez,[1,3] Lance A. Mulder,[2] Vivi M. Heine,[4,5] Pavlina Konstantinova,[1] Sander J. van Deventer,[1,3] Katja C. Wolthers,[2] Dasja Pajkrt,[6] Adithya Sridhar,[2] and Melvin M. Evers[1]

[1]Department of Research & Development, uniQure Biopharma B.V., Amsterdam, the Netherlands; [2]Department of Medical Microbiology, Laboratory of Clinical Virology, Amsterdam UMC, Amsterdam, the Netherlands; [3]Department of Gastroenterology and Hepatology, Leiden University Medical Center, Leiden, the Netherlands; [4]Department of Complex Trait Genetics, Center for Neurogenomics and Cognitive Research, Amsterdam Neuroscience, Vrije Universiteit, Amsterdam, the Netherlands;; [5]Department of Child & Youth Psychiatry, Emma Children's Hospital, Amsterdam UMC, Amsterdam, the Netherlands; [6]Department of Pediatric Infectious Diseases, Emma Children's Hospital, Amsterdam UMC, Amsterdam, the Netherlands

The development of gene therapies for central nervous system disorders is challenging because it is difficult to translate preclinical data from current *in vitro* and *in vivo* models to the clinic. Therefore, we developed induced pluripotent stem cell (iPSC)-derived cerebral organoids as a model for recombinant adeno-associated virus (rAAV) capsid selection and for testing efficacy of AAV-based gene therapy in a human context. Cerebral organoids are physiological 3D structures that better recapitulate the human brain compared with 2D cell lines. To validate the model, we compared the transduction efficiency and distribution of two commonly used AAV serotypes (rAAV5 and rAAV9). In cerebral organoids, transduction with rAAV5 led to higher levels of vector DNA, transgenic mRNA, and protein expression as compared with rAAV9. The superior transduction of rAAV5 was replicated in iPSC-derived neuronal cells. Furthermore, rAAV5-mediated delivery of a human sequence-specific engineered microRNA to cerebral organoids led to a lower expression of its target ataxin-3. Our studies provide a new tool for selecting and deselecting AAV serotypes, and for demonstrating therapeutic efficacy of transgenes in a human context. Implementing cerebral organoids during gene therapy development could reduce the usage of animal models and improve translation to the clinic.

INTRODUCTION

Gene therapy has the potential to treat inherited diseases, such as genetic neurodegenerative disorders, for which currently no treatment is available. One of the most commonly used vectors to safely and effectively deliver therapeutic transgenes to patients is non-pathogenic recombinant adeno-associated viruses (rAAVs).[1] Due to variations in the AAV capsids, AAV serotypes have different cell tropism and transduction efficiencies.[2,3] To improve the specificity and transduction efficiency of rAAVs in the brain, new rAAV variants are engineered by directed evolution of existing AAV capsids.[4,5] However, the translation from preclinical models to effective drugs in humans remains a challenge for gene therapy development, and this is a major issue for the development of central nervous system (CNS)-targeted therapies.[6] This is illustrated by the engineered AAV9-Php.b, a variant of AAV9, which showed great blood-brain barrier penetration in a mouse model. However, this was mouse strain specific, and further clinical development was halted.[7,8]

The limited success rate of translation to the clinic is partly due to the complexity and inaccessibility of the brain and limitations of traditional *in vitro* and *in vivo* preclinical CNS models that are used for AAV capsid and transgene optimization. Traditionally transformed neural-like cell lines that are used as *in vitro* models display aberrant expression profiles. Cell lines are grown in 2D and therefore lack complex cell-cell interactions and cell-extracellular matrix interactions, which both have an effect on cell polarity, differentiation, and proliferation.[9] *In vivo* models can model complex cellular microenvironments and systemic interactions but often lack human-specific features, such as permissiveness to the vector and human CNS-specific genetics, which are important for testing transgene efficacy and safety. Additionally, *in vivo* research has low throughput, is expensive, and should also be minimized from an ethics standpoint. New innovations in experimental modeling of the brain are therefore needed.

The development of human cerebral organoids has quickly advanced and provides new opportunities for translating preclinical studies to the clinic in brain disease.[9] Cerebral organoids are 3D cell cultures, harboring different neural cell types and brain regions, derived from either human embryonic stem cells or human induced pluripotent stem cells (iPSCs).[10] Cerebral organoids more closely resemble the

Received 24 March 2020; accepted 27 May 2020;
https://doi.org/10.1016/j.omtm.2020.05.028.

Correspondence: M.M. Evers, Department of Research & Development, uniQure Biopharma B.V., Amsterdam, the Netherlands.
E-mail: m.evers@uniqure.com

© 2020 The Author(s).
This is an open access article under the CC BY license (http://creativecommons.org/licenses/by/4.0/).

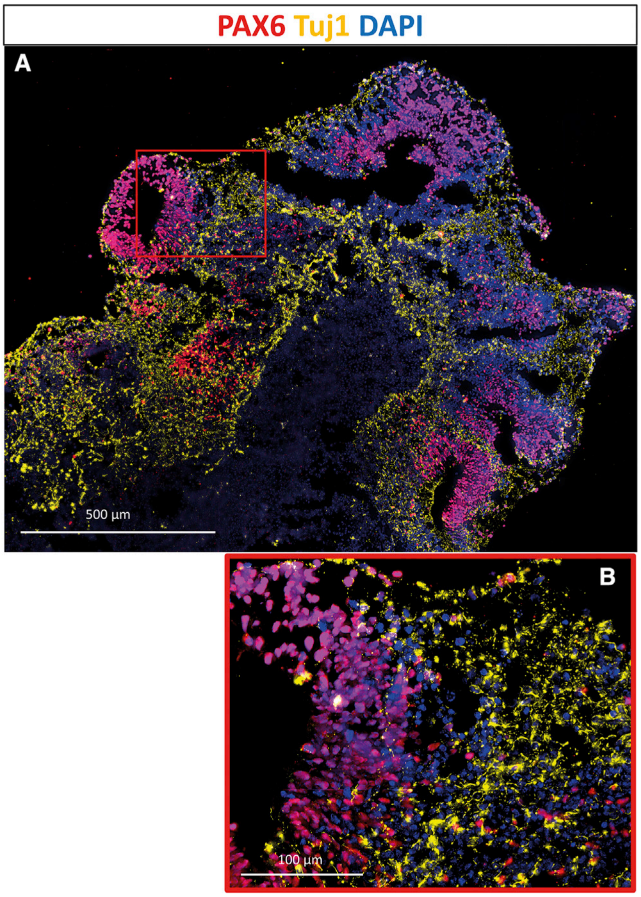

Figure 1. Characterization of Cerebral Organoids via Immunostaining

(A and B) Representative image (A) and excerpt (B) of a section of a cerebral organoid after 30 days, showing a structure similar to the fetal brain. Neural progenitor nuclei were stained with anti-PAX6 (red) and neuron cell bodies with anti-Tuj1 (yellow) antibodies. Nuclei were visualized with DAPI (blue). (A) scale bar is 500 μm, (B) scale bar is 100 μm.

human *in vivo* situation than 2D cell lines with respect to differentiation, organization, and polarity, and have higher throughput compared with animal models.[9,11] As a result of these advantages, human disease models have been generated for neurodegenerative diseases that lack predictive animal models, such as Alzheimer's disease, Parkinson's disease, motor neuron disease, and frontotemporal dementia.[12] Additionally, cerebral organoids have been valuable for studying neurodevelopment and difficult-to-model neurotropic viruses, such as Zika virus, herpes simplex virus, Japanese encephalitis virus, and dengue virus,[13–16] or as a screening platform for drug discovery.[17] Comparably, retinal organoids, which are similar to cerebral organoids in their differentiation method, have been used to compare AAV serotypes for retinal gene therapy, suggesting the prospect for testing gene therapy approaches in brain organoids.[3,18] Finally, cerebral organoids have been shown to be susceptible to an AAV9 variant that targets astrocytes, and cerebral organoids were used to test the efficacy of an AAV9-based gene therapy for GM1 gangliosidosis.[19,20] Together, these findings indicate the potential of using cerebral organoids to study AAV-mediated transduction in the human brain.

In this study, we validate cerebral organoids as a model for AAV-mediated transduction of the brain. We demonstrate that cerebral organoids can be used to select the AAV capsid with the highest transduction efficiency. We show that cerebral organoids can be used as a model for proof of concept of transgene efficacy studies in the human brain. For both of these important applications during development of novel gene therapies, a relevant human model is key.

RESULTS

Generation of Cerebral Organoids

In order to validate cerebral organoids as a model for gene therapy in the CNS, we generated and characterized cerebral organoids from human iPSCs derived from a healthy control. At day 30, the organoids formed cavities, resembling ventricles, with surrounding neural progenitor cells (NPCs) positive for NPC marker PAX6 (Figure 1A). The NPC-rich regions around ventricles were also positive for proliferation marker Ki67 (Figure S1). Adjacent to the proliferating NPCs, a layer of neurons was positive for neural markers Tuj1 (Figure 1B) and MAP2 (Figure S1). The organoids were negative for neural crest cell marker SOX10 and pluripotency marker OCT4, which means that the organoids did not retain undifferentiated cells or neural crest cells. This characterization of our organoids is in line with the previously described organoids using the same protocol.[10]

Comparing rAAV5 and rAAV9 Transduction Efficiency in Cerebral Organoids

To validate cerebral organoids as a translatable model for AAV capsid selection, we transduced 66-day-old cerebral organoids with rAAV5 and rAAV9, which are used in clinical trials (ClinicalTrials.gov: NCT03315182 and NCT04120493), both carrying a transgene that expresses green fluorescent protein (GFP) under the control of the chicken β actin (CAG) promotor. By confocal imaging of immunostained organoid sections, we observed strong *GFP* expression of the organoids (Figure 2). Because the AAV was administered to the organoid medium and not injected into the organoids, we expected the transduced cells on the outer layer of the organoids. However, cells expressing *GFP* were found in the center of the organoid as well. To quantify the transgene expression, we measured the mean GFP intensity in multiple z stacks per organoid section and normalized it to the DAPI signal. The transgene intensity was not uniform across the organoid section, and there was variation between organoids. Despite the variation, a significantly higher GFP intensity (2.2-fold) was measured in rAAV5-transduced organoids compared with rAAV9-transduced organoids (n = 3 organoids of one batch; $p < 0.005$) (Figure S2).

To accurately quantify the differences in transduction efficiency, we transduced cerebral organoids with rAAV5 and rAAV9 carrying a gene expressing secreted alkaline phosphatase (SEAP) under the control of the cytomegalovirus (CMV) promotor. Because SEAP is a secreted enzyme, we determined the transduction efficiency by

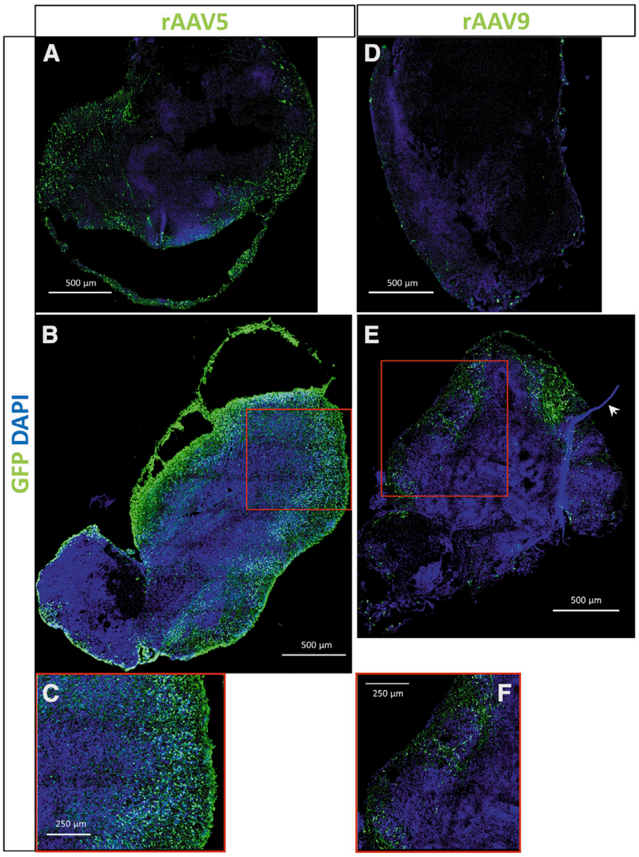

Figure 2. AAV Transduction in Cerebral Organoids

(A,B,D,E) Representative images of two organoids transduced with rAAV5-GFP- (A,B) and two organoids transduced with rAAV9-GFP- (D,E) show expression of *GFP* transgene with both serotypes. GFP was stained with αGFP antibody, and nuclei were dyed with DAPI. The images are a maximum projection of a tile scan of z stacks acquired by confocal imaging. (E) Arrowhead shows a suture fragment that is added during organoid formation for improved morphology (see Materials and Methods). (C) and (F) are zoomed-in regions of (B) and (E), respectively. (A,B,D,E) scale bar is 500 µm, (C,F) scale bar is 250 µm.

measuring the SEAP activity in medium samples taken over time. Cell entry, uncoating, transgene expression, and translation appeared to have already taken place within the first 24 h, because rAAV5-transduced organoids showed SEAP activity at 1 day post-transduction (Figure 3A). rAAV5 transduction led to high SEAP activity levels in the medium after 3 days, which was 38 times higher compared with rAAV9 treatment (n = 9; three batches with three organoids per condition). On day 3, the medium of the organoids was refreshed, and on days 6 and 7, the SEAP activity in the medium of rAAV5 and rAAV9 organoids further increased, while the fold change between rAAV5 and rAAV9 remained similar (43-fold on day 6 and 38-fold on day 7).

To support these clear differences in transduction efficiency between rAAV5 and rAAV9, we measured vector DNA (vDNA) and *SEAP* mRNA expression by qPCR in rAAV5- and rAAV9-treated organoids. rAAV5-SEAP-transduced organoids contained 358 (±263 SD) copies of vDNA per cell, which is 5.3 times more compared with rAAV9-transduced organoids (Figure 3B). In addition, the *SEAP* mRNA was measured in 13.1 times higher quantities in rAAV5-transduced organoids compared with rAAV9 (Figure 3C). This supports previous immunostaining results confirming that, in this cerebral organoid model, rAAV5 transduces neuronal cells more efficiently compared with rAAV9.

Comparing rAAV5 and rAAV9 Transduction Efficiency in 2D iPSC-Derived Neuronal Cultures

To test the congruency of the cerebral organoids with another *in vitro* model of the brain, we compared the results from the cerebral organoids with transduction efficiencies in iPSC-derived neural cells grown in 2D. rAAV5 transduction led to higher levels of vDNA compared with rAAV9-SEAP in neuronal cells transduced at three different doses (1×10^{10}, 1×10^{11}, and 1×10^{12} genome copies [gc]/well) (Figure 4A). However, this difference was significant only at the highest dose. The more efficient transduction by rAAV5 was also reflected in higher SEAP activity in the medium compared with rAAV9 (Figure 4B). The difference between rAAV5 and rAAV9 was less pronounced at higher doses based on SEAP activity, whereas the opposite was true based on vDNA levels. The smaller fold change in SEAP activity in higher doses could be the result of transgene expression reaching a plateau. At doses of 10^{10} and 10^{11} gc/well, the fold change of rAAV5/AAV9 was higher in transgene activity (27.2- and 5.3-fold) compared with vDNA (1.6- and 1.9-fold), which is in line with the findings in cerebral organoids.

Modeling Transgene Efficacy in Cerebral Organoids

To demonstrate the feasibility of cerebral organoids as a translatable model for transgene efficacy and target engagement, we tested the efficacy of an rAAV5-based candidate for the treatment of spinocerebellar ataxia 3 (SCA3) in cerebral organoids. In SCA3, a CAG repeat in the *ATXN3* gene causes accumulation of mutant ataxin-3 protein in the brain, leading to neurodegeneration.[21] We engineered an AAV-delivered microRNA (miATXN3) that targets the *ATXN3* mRNA, thereby preventing accumulation of the ataxin-3 protein. We measured high numbers of rAAV5-miATXN3 vDNA (94 gc/cell ± 48 SD) by qPCR in transduced organoids 9 days after transduction, but not in rAAV5-GFP- or mock-transduced organoids (Figure 5A). In addition, rAAV5-miATXN3 transduction led to the expression of on average 13 *miATXN3* molecules per cell (±11 SD; Figure 5B). The miRNA expression led to a 30% lower expression of ataxin-3 protein in transduced organoids compared with control organoids as measured by time-resolved fluorescence energy transfer (TR-FRET). The rAAV5-GFP transduction did not result in a lowering, proving that *miATXN3*, and not the AAV transduction, lowered the expression of ataxin-3 in cerebral organoids.

DISCUSSION

The goal of this study was to develop a translatable human organoid system to evaluate efficacy of gene therapy in neurodegenerative diseases. We investigated whether cerebral organoids are suitable

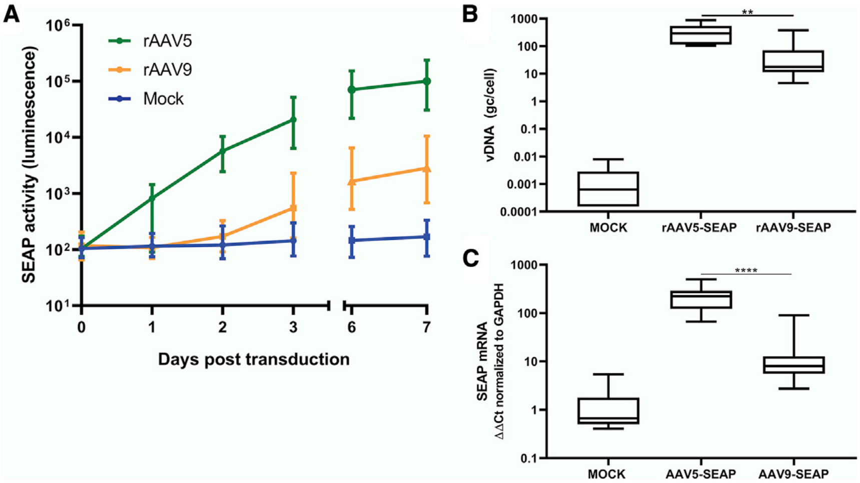

Figure 3. Comparing rAAV5 and rAAV9 Transduction Efficiency in Cerebral Organoids Based on Transgene Activity, vDNA, and Transgene mRNA Expression

(A) Transgene SEAP activity levels were higher in rAAV5-transduced organoids compared with rAAV9-transduced organoids. Medium was refreshed after 3 days. On 0, 1, 2, 3, 6, and 7 days after transduction, medium samples were taken from transduced organoids. SEAP activity was measured via a luminescence assay. Three days after transduction, the SEAP activity level was 38 times higher in rAAV5-transduced organoids compared with rAAV9 (n = 3 batches of 3 organoids each, graph depicts mean with interquartile range). (B) In rAAV5-transduced organoids, 5.3 times higher vDNA levels were measured by qPCR compared with rAAV9 (n = 3 batches of 3 organoids each; two-way ANOVA followed by Tukey's multiple comparison, **$p < 0.005$; graphs represent mean and min to max values). (C) mRNA *SEAP* expression in organoids was significantly higher, 13.1 times, in organoids transduced by rAAV5 compared with rAAV9. mRNA *SEAP* levels in RNA isolates of organoids were measured by qPCR and normalized to GAPDH (n = 3 batches of 3 organoids each; two-way ANOVA followed by Tukey's multiple comparison, ****$p < 0.0001$; graphs represent mean and min to max values).

for modeling differences between AAV serotypes (rAAV5 and rAAV9) and have utility to study the efficacy of therapeutic lowering of human ataxin-3 by rAAV5-miATXN3.

Our results showed a higher transduction efficiency of rAAV5 compared with rAAV9 in cerebral organoids. This finding was consistent across experiments with two different transgenes and using both CMV and CAG constitutive promotors. rAAV5 transduction led to higher levels of vDNA and transgene mRNA and protein compared with rAAV9. Interestingly, the fold change in mRNA was higher compared with the fold change in vDNA. This suggests that, in the brain cells tested, rAAV5 is not only more efficient in entering the cells but also in downstream processes, such as capsid uncoating, endosomal escape, and/or nuclear entry.[22,23] The improved transduction efficiency of rAAV5 over rAAV9 was replicated in neuronal cultures derived from iPSCs of a different donor. Imaging of transduced organoids revealed that not only the outer layer of the organoid is transduced, but transgene was even expressed in the center of the organoid, which may have occurred through anterograde and retrograde transport that has been reported for both rAAV5 and rAAV9.[24–26]

In human patients, rAAV5 and rAAV9 have both been proved to be effective as a delivery system for treating neurological disorders.[27–29] The transduction efficiency of both vectors, however, has been directly compared only in preclinical studies, with conflicting results. One factor that could explain the difference in results is the use of various administration routes. For instance, AAV9 transduction has been shown to be more efficient compared with AAV5 transduction after injection into the thalamus,[30] hippocampus, or auditory cortex, whereas AAV5 transduction of the brain has been shown to be more widespread after injection in the striatum[31,32] or spinal cord.[33] For AAV9, intravenous injection is a known alternative administration route for CNS transduction.[34] As mentioned earlier, the model species also affects transduction efficiencies.[7,8,32] Besides the model species and administration route, differences in AAV production methods and the dosing could also have affected transduction

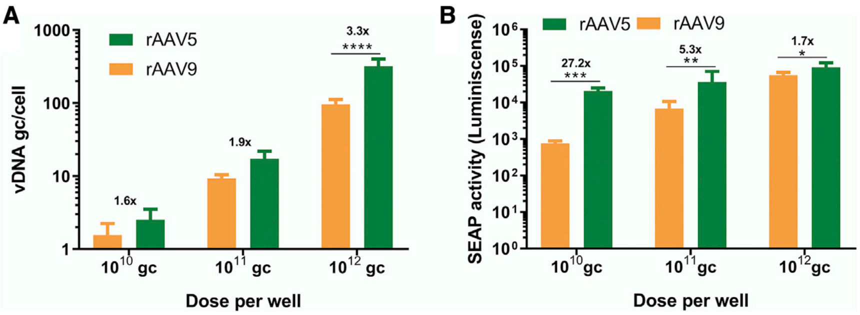

Figure 4. Comparing rAAV5 and rAAV9 Transduction Based on vDNA and SEAP Transgene Activity in 2D Neuronal Cultures

(A) rAAV5-SEAP transduction of 2D iPSC-derived neuronal cultures led to higher levels of vDNA compared with rAAV9-SEAP as measured by qPCR. Only at a dose of 10^{12} genome copies (gc)/well was the increase significant (n = 3 wells; two-way ANOVA, Sidak's multiple comparison, ****$p < 0.0005$; mean with 95% CI). (B) rAAV5-SEAP transduction led to a significantly increased activity of transgenic SEAP compared with rAAV9-SEAP in all three doses tested, as measured by a luminescence assay (n = 3 wells; two-way ANOVA, Sidak's multiple comparison, *$p < 0.05$, **$p < 0.005$, ***$p < 0.0005$; mean with 95% CI). The difference between the serotypes was the most pronounced at lower doses.

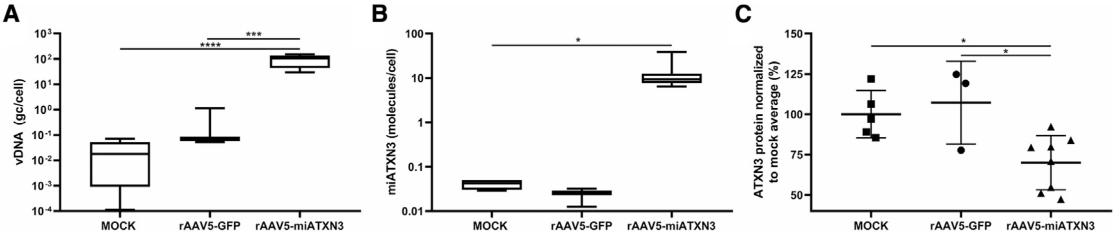

Figure 5. Measuring the Effect of a Therapeutic Transgene in rAAV5-Transduced Cerebral Organoids

(A) rAAV5-CAG-miATXN3 vDNA was measured by qPCR and depicted as gc per cell. rAAV5-miATXN3-transduced organoids contained on average 94 gc of vDNA/cell (rAAV5-miATXN3: n = 3 batches of 3 organoids each, Mock: n = 2 batches of 3 organoids each, rAAV5-GFP: n = 1 batch of 3 organoids; one-way ANOVA followed by Tukey's multiple comparison, ***$p < 0.0005$, ****$p < 0.00005$; graphs represent mean and min to max values). (B) miRNA was measured in cerebral organoids by qPCR. rAAV5-miATXN3-transduced organoids expressed on average 13 miRNA copies per cell (see A; n = 3; one-way ANOVA followed by Tukey's multiple comparison, *$p < 0.05$; graphs represent mean and min to max values). (C) Ataxin-3 protein was measured by TR-FRET in lysates of cerebral organoids transduced by rAAV5-miATXN3, or control-transduced organoids expressed 30% less ataxin-3 protein as measured by TR-FRET (see A; n = 3; one-way ANOVA followed by Tukey's multiple comparison, *$p < 0.05$; graphs represent mean with SD).

efficiency. In our study, both rAAV5 and rAAV9 were produced in Sf$^+$ cells with a baculovirus-based production system. The vector production system may affect infectivity of AAV serotypes, and therefore the current data should not be generalized to indicate the superiority of AAV5 over AAV9 for transducing the brain. However, the fact that significant differences between two serotypes were repeatedly measured in the cerebral organoid model and that the results could be reproduced in 2D iPSC-derived neuronal cultures indicates that cerebral organoid is an easy-to-access and reproducible model to study transduction efficiency for capsid selection in human studies.

Next, we investigated the potential of using cerebral organoids to measure the efficacy of a gene therapy for genetic brain disorder SCA3. It has been shown that in iPSC-derived neurons and SCA3 mice, transduction with rAAV5-miATXN3 leads to lowering of ataxin-3 protein.[35,36] This was measured using a robust TR-FRET immunoassay. With the same protein quantification assay, we measured a 30% lowering of ataxin-3 protein levels in cerebral organoids after rAAV5-miATXN3 transduction and subsequent *miATXN3* expression. The protein lowering was due to the miRNA-based targeting and not the transduction itself, because rAAV5-GFP did not affect the ataxin-3 levels. Reduction of ataxin-3 per transduced cell was likely higher because the reduction was diluted by non-transduced cells in the center of the organoid. Therefore, it would be an improvement to quantify the percentage of transduced cells. Additionally, multiple doses could be tested to further assess the efficacy of the transgene. As a proof of concept, our data showed that human iPSC-derived cerebral organoids are able to model the therapeutic effect of an AAV-delivered transgene.

Comparing cerebral organoids with current preclinical models, the 3D complexity of cerebral organoids gives the disadvantage of increased heterogeneity over neuronal cell line cultures.[37] Although our organoids showed variability in, for example, ataxin-3 levels, the results were consistent across multiple batches of organoids. A major advantage of human cerebral organoids over animal models is their genetic background. This is advantageous in testing efficiency and specificity of promotors, and makes it possible to use them for assessing human-specific safety of therapeutic transgenes and formulations. Besides the benefit of the human context in general, the iPSC technology enables the generation of patient-specific organoids. Patient-specific organoids have been useful for testing therapeutic efficacy in a human disease model where human primary tissue is inaccessible or disease models do not fully represent the human disease phenotype[20] and could be useful for personalized medicine as well.[16,38] Incorporating disease phenotypes in cerebral organoids could potentially reduce, replace, or circumvent the use of diseased rodent models. However, although cerebral organoids are more complex than 2D *in vitro* models, they do not fully represent the complexity of *in vivo* models. Although progress is made to generate even more complex cerebral organoids,[39,40] they still lack important features, such as vasculature, the blood-brain barrier, and a relevant immune system, and do not represent the scale of the human brain. Therefore, animal models are still needed to study the role of these features in gene therapy. Finally, cerebral organoids are better scalable and generated faster compared with animal models. As we demonstrated, AAV-mediated transduction can be frequently monitored by testing the cultured medium for secreted transgenes, which is an advantage over animal models for CNS diseases.

In conclusion, our studies provide a new tool for selecting and deselecting AAV serotypes and for demonstrating therapeutic efficacy of transgenes. Both of these applications are crucial during the preclinical development of novel gene therapies and could benefit from a human *in vitro* model of the brain. Implementing cerebral organoids during gene therapy development could reduce the usage of animal models and improve translation to the clinic.

MATERIALS AND METHODS

iPSCs and Organoid Generation

Human iPSCs, kindly provided by Vivi Heine (Amsterdam UMC, the Netherlands) were cultured in vitronectin (STEMCELL Technologies, Vancouver, BC, Canada)-coated six-well plates and in mTesr$^+$ medium (STEMCELL Technologies, Vancouver, BC, Canada). iPSCs tested

negative for karyotypic abnormalities by G-band analysis (data not shown) before differentiation. From these iPSCs, cerebral organoids were made using the cerebral organoid kit from STEMCELL Technologies, which is based on the protocol described by Lancaster and Knoblich.[10] In short, iPSCs were harvested with gentle cell dissociation reagent (STEMCELL Technologies, Vancouver, BC, Canada). The single cells were then seeded in a round-bottom ultra-low-attachment plate (Corning, Corning, NY, USA) containing around five suture fragments of ±1 mm polyamide, size 4-0 (Ethicon, Cincinnati, OH, USA). In 5 days, embryonic bodies (EBs) formed along the suture fragments. Neuroectoderm formation was induced by transferring the EBs to induction medium (STEMCELL Technologies, Vancouver, BC, Canada). Subsequently, the EBs were embedded in Matrigel (STEMCELL Technologies, Vancouver, BC, Canada) droplets. Expansion medium (STEMCELL Technologies, Vancouver, BC, Canada) was added in order to let the neuroepithelium expand and form neuroepithelial buds. Finally, the organoids were matured in organoid maturation medium in a six-well plate while shaking on an orbital shaker (INFORS, Bottmingen, Switzerland) at 66 rpm at 37°C and 5% CO_2. With this protocol, three batches of organoids were made from the same iPSC line but with different passage numbers. At day 30, organoids were characterized via immunostaining.

iPSC-Derived Neuronal Cells

2D neuronal cultures were differentiated from ND42229*B iPSCs as described by Keskin et al.[41] ND42229*B is an iPSC line derived from Huntington's patient fibroblasts. iPSCs were cultured in poly(D) and laminin-coated 24-well plates. Neural differentiation was performed by dual SMAD inhibition according to the STEMCELL Technologies kit and yielded a mixture of neuronal and astrocytic cells. The cells were cultured in BrainPhys medium (STEMCELL Technologies, Vancouver, BC, Canada) before transduction.

Virus Production

rAAVs were generated in a baculovirus-based AAV production system as described previously.[35,42] Sf^+ cells were triple infected with three different recombinant baculoviruses expressing the transgene (*GFP*, *SEAP*, or *miATXN3*) and promotor (CMV or CMV early enhancer/CAG) flanked by inverted terminal repeats, the replicon enzyme, and the capsid protein for either rAAV5 or rAAV9, to generate rAAV5-CAG-GFP, rAAV9-CAG-GFP, rAAV5-CMV-SEAP, rAAV9-CMV-SEAP, and rAAV5-CAG-miATXN3. The Sf^+ cells were lysed 72 h after the triple infection, and the crude lysate was treated with 8 U/mL Benzonase (Merck, Darmstadt, Germany) for 1 h at 37°C. rAAV5 was purified on an AVB Sepharose column (GE Healthcare, Little Chalfont, UK) and rAAV9 on POROS CaptureSelect AAV9 (Thermo Fisher, Waltham, MA, USA), both using an AKTA purification system (GE Healthcare, Chicago, IL, USA). The final titer concentration was determined by qPCR.

AAV Transduction

Cerebral organoids of 66 days old were transduced with rAAV5-CAG-GFP, rAAV9-CAG-GFP, rAAV-CMV-SEAP, rAAV9-CMV-SEAP, and rAAV5-CAG-miATXN3. The organoids were transferred to an ultra-low-attachment 96-well plate (Corning, New York, NY, USA). 35 μL of 6×10^{11} gc of rAAV in phosphate-buffered saline (PBS) 5% sucrose was added to each organoid. After 1-h incubation at 37°C 5% CO_2, the organoids were transferred to a well containing 3 mL organoid maturation medium (STEMCELL Technologies, Vancouver, BC, Canada) in a six-well plate and incubated at 37°C 5% CO_2 while shaking at 66 rpm for 9 days. Medium was changed on days 3 and 7. 2D neuronal cell cultures were seeded in a 24-well plate at a density of 10^5 cells/well. 10^{10}, 10^{11}, or 10^{12} gc of rAAV5-CMV-SEAP or rAAV9-CMV-SEAP per well in 100 μL 5% PBS and 400 μL BrainPhys (STEMCELL Technologies, Vancouver, Canada) was added. After 2 days the medium was changed.

Sectioning and Staining

Organoids were fixed in 2 mL 10% formalin at room temperature for 1 h. Next, to protect against freezing artifacts, the organoids were incubated in 5 mL 30% sucrose in PBS overnight. After cryopreservation, the organoids were frozen and embedded in Tissue Tek optimal cutting temperature compound (OCT) (Sakura Finetek EU, Alphen aan den Rijn, the Netherlands), directly on dry ice, and stored at −80°C until further processing. For characterization of organoids, 8-μm sections were cut; for rAAV5-rAAV9 comparison, 20-μm sections were cut by using a CryoStar NX70 cryostat (Thermo Fisher Scientific, Waltham, MA, USA). Sections were placed on Superfrost plus slides (Thermo Fisher Scientific, Waltham, MA, USA) and stored at −30°C. The sections were permeabilized and blocked with 10% bovine serum albumin 0.5% Tween in PBS for 2 h. Sections were stained with PAX6, Nestin, OCT4, Tuj1 (STEMCELL Technologies, Vancouver, BC, Canada), SOX10, ki67 (Abcam, Cambridge, UK), and MAP2 (Invitrogen, Carlsbad, CA, USA) primary antibodies overnight. After washing, secondary antibodies (1:750 goat α-mouse Alexa Fluor 488 (AF488), goat α-rabbit AF568, and goat α-chicken AF647; Thermo Fisher Scientific, Waltham, MA, USA) were added, and the slides were incubated for 1 h at 4°C. Nuclei were stained with DAPI (Invitrogen, Carlsbad, CA, USA) and mounted with Prolong Gold antifade reagent (Invitrogen, Carlsbad, CA, USA).

SEAP Activity Assay

SEAP transgene expression in transduced cerebral organoids and 2D neuronal cell cultures was determined by measuring the enzyme activity in the medium with the SEAP reporter gene assay (Roche, Basel, Switzerland). On 0, 1, 2, 3, 5, and 6 days after organoid transduction, 60 μL medium was sampled. Per measurement, 10 μL sample was diluted 20 times. Four days after 2D neuronal cell transduction, medium was sampled and diluted four times. Diluted samples of organoid and 2D neuronal cell medium were heat inactivated at 60°C to inactivate endogenous alkaline phosphatases. After adding SEAP substrate to the sample in duplicate, the luminescence was measured for 1 s on a GloMax Discover microplate reader (Promega, Madison, WI, USA).

DNA and RNA

Organoids were snap frozen in liquid nitrogen and stored at −80°C. Frozen rAAV5-CAG-miATXN3-transduced organoids and respective controls were cut in two with a scalpel without thawing. For

mRNA, miRNA, and vDNA analysis, half of cut organoids and whole AAV-SEAP-transduced organoids were homogenized in a gentleMACS Octo Dissociator (Miltenyi Biotec, Bergisch Gladbach, Germany) or FastPrep-24 5G (MP Biomedicals, Irvine, CA, USA) in 600 μL TRIzol (Invitrogen, Carlsbad, CA, USA). The samples were then centrifuged at 10,000 × *g* for 30 s. Total RNA and vDNA were isolated with Direct-zol kit (Zymo Research, Irvine, CA, USA). RNA yield was measured with NanoDrop 2000 (Thermo Fisher Scientific, Waltham, MA, USA). vDNA was measured in the RNA isolates via RT-PCR. For AAV5-CMV-SEAP and AAV9-CMV-SEAP vDNA, primers specific for the CMV promoter sequence were used. For AAV5-CAG-miATXN3 vDNA, poly(A) signal-specific primers were used. The total vDNA was calculated based on a plasmid standard line and reported as gc per cell based on 15 pg total RNA per cell.[43] For *SEAP* mRNA expression measurement, cDNA synthesis was performed using the Maxima First Strand cDNA synthesis kit (Thermo Fisher Scientific, Waltham, MA, USA) with random hexamer primers. Non-reverse-transcriptase controls were taken along. mRNA was measured via RT-PCR with TaqMan primers specific for the *SEAP* sequence (Thermo Fisher Scientific, Waltham, MA, USA). The mRNA expression levels were normalized to human glyceraldehyde-3-phosphate dehydrogenase (GAPDH; Custom TaqMan small RNA assays, assay ID: Hs02758991 g1; Thermo Fisher Scientific, Waltham, MA, USA). *ATXN3* miRNA (miATXN3) expression was measured by a customized TaqMan RT-PCR assay (Custom TaqMan small RNA assays, Assay ID: CTEPRZE; Thermo Fisher Scientific, Waltham, MA, USA).

Protein

For ataxin-3 protein analysis, the other half of the cut organoids were homogenized in FastPrep-24 5G (MP Biomedicals, Irvine, CA, USA) in 1% Triton X-100. Lysates were centrifuged at 10,000 × *g* for 30 s, incubated for 30 min at 4°C at 300 rpm before spinning down at 10,000 × *g* for 10 min. Total protein concentration was determined with a bicinchoninic assay. Ataxin-3 protein was measured by TR-FRET. Antibody labeling was done after dialyzing, in order to adjust pH. α-Rb-α-ataxin-3 (Abcam, Cambridge, UK) was labeled with terbium cryptate as a donor fluorophore at pH 8.0. α-Ataxin-3 clone 1H9 (Millipore, Burlington, MA, USA) was labeled with d2 as an acceptor fluorophore at pH 9.0. Terbium detection buffer, labeled antibodies, and protein lysate were added to a 384-well plate. The plate was sealed and incubated at 4°C overnight. Before measurement, the plate was set to reach room temperature. Fluorescence was read on a Synergy H1 plate reader (Biotek, Winooski, VT, USA) using a filter-based detection mode at 620 nm to measure donor fluorescence and at 665 nm to measure acceptor fluorescence. Protein concentration was calculated by dividing the ratio between donor and acceptor fluorescence signal (ΔR) by the ΔR of a PBS control.

Imaging and Analysis

For characterization of cerebral organoids, slides were imaged using a AXIO Z1 scan slide scanner (Zeiss, Oberkochen, Germany). As a light source, the solid-state light source Colibri 7 was used with a red (630 nm) light-emitting diode (LED) for Cy5, a green (555 nm) LED for the AF568, a blue (475 nm) LED for the AF488, and a UV (385 nm) LED for DAPI. For all fluorochromes, the multi-band filter cube 90 high efficiency was used with an emission filter of 425/30 for DAPI, 514/30 for AF488, 592/30 for Cy3, and 709/100 for Cy5. The detection of the emitted light was acquired with the 16-bit High-End monochromatic Orca Flash 4.0 V3 camera with 2,048 × 2,048 pixels and 6.5-μm pixel size. Course focusing was performed with a 10× plan-apochromatic objective with 0.45 numerical aperture (NA) while fine focusing, and image acquisition was performed with the use of a 40× plan-apochromat objective with 0.95 NA. For each slide the whole organoid was imaged with five z stacks above and below the detected focus plane of 1-μm steps. The single images were stitched to one czi-file in the online-processing mode of the Zeiss software ZEN blue edition v.2.6.7.6.00000. After image acquisition, the Zeiss software ZEN blue edition V3.0.79.00004 was used to create an extended depth of focus image from the z stack using the maximum projection function.

For imaging transduced organoids, a tile scan of a whole organoid section was acquired with the 40× objective, using a SP8-X confocal microscope (Leica, Wetzlar, Germany). From each section of three regions, a z stack (15 steps of 0.75 μm) was acquired with the 40× objective and zoom of 2.5. The regions were picked on a fixed 1-μm distance from the edge of the section. From three organoids from the same batch, two sections were acquired per organoid. From each z stack the average GFP intensity was measured in LasX software (Leica, Wetzlar, Germany). As a control, a z stack was made in the center of the section, where no GFP-positive cells were visible. The GFP signal of this control z stack was subtracted from the GFP intensities as background. The GFP signal was then normalized to the average DAPI intensity of the z stack to control for a difference in cell number. Images were processed with ImageJ (version 1.52a).

SUPPLEMENTAL INFORMATION

Supplemental Information can be found online at https://doi.org/10.1016/j.omtm.2020.05.028.

AUTHOR CONTRIBUTIONS

Conceived and designed the experiments: J.A.D., M.S.-G., L.M., A.S., M.M.E.; performed the experiments: J.A.D., M.S.-G., L.M.; analyzed the data: J.A.D., M.S.-G., L.M.; contributed the iPSCs: V.M.H.; wrote the manuscript: J.A.D., A.S., M.M.E.; supervision: K.C.W., D.P.; funding: P.K., S.J.v.D.

CONFLICTS OF INTEREST

J.A.D., M.S.-G., M.M.E., P.K., and S.J.v.D. are employees and/or shareholders of uniQure B.V.

ACKNOWLEDGMENTS

We thank Fanny Martier, Cynthia Brouwers, and Sonay Keskin for helping with the qPCR and TR-FRET experiments, and Bas Bosma, Francois Du Plessis, and Jacek Lubelski for providing the AAVs. We thank Lukas Schwarz, Sumiati Baatjes, and Valerie Sier-Ferreira

for input and assisting with staining and imaging of the cerebral organoids; Ron Hoeben and Daisy Picavet for helping with the analysis of the confocal images; Ellen Broug and Sietske Gadella for critical reading of the manuscript; and all group members for helpful discussions. This research received funding by European Union's Horizon 2020 innovative training network under grant number 812673 (OrganoVIR).

REFERENCES

1. Choudhury, S.R., Hudry, E., Maguire, C.A., Sena-Esteves, M., Breakefield, X.O., and Grandi, P. (2017). Viral vectors for therapy of neurologic diseases. Neuropharmacology *120*, 63–80.
2. Hammond, S.L., Leek, A.N., Richman, E.H., and Tjalkens, R.B. (2017). Cellular selectivity of AAV serotypes for gene delivery in neurons and astrocytes by neonatal intracerebroventricular injection. PLoS ONE *12*, e0188830.
3. Gonzalez-Cordero, A., Goh, D., Kruczek, K., Naeem, A., Fernando, M., Kleine Holthaus, S.M., Takaaki, M., Blackford, S.J.I., Kloc, M., Agundez, L., et al. (2018). Assessment of AAV Vector Tropisms for Mouse and Human Pluripotent Stem Cell-Derived RPE and Photoreceptor Cells. Hum. Gene Ther. *29*, 1124–1139.
4. Sun, S., and Schaffer, D.V. (2018). Engineered viral vectors for functional interrogation, deconvolution, and manipulation of neural circuits. Curr. Opin. Neurobiol. *50*, 163–170.
5. Kotterman, M.A., and Schaffer, D.V. (2014). Engineering adeno-associated viruses for clinical gene therapy. Nat. Rev. Genet. *15*, 445–451.
6. Gribkoff, V.K., and Kaczmarek, L.K. (2017). The Need for New Approaches in CNS Drug Discovery: Why Drugs Have Failed, and What Can Be Done to Improve Outcomes. Neuropharmacology *120*, 11–19.
7. Hordeaux, J., Wang, Q., Katz, N., Buza, E.L., Bell, P., and Wilson, J.M. (2018). The Neurotropic Properties of AAV-PHP.B Are Limited to C57BL/6J Mice. Mol. Ther. *26*, 664–668.
8. Liguore, W.A., Domire, J.S., Button, D., Wang, Y., Dufour, B.D., Srinivasan, S., and McBride, J.L. (2019). AAV-PHP.B Administration Results in a Differential Pattern of CNS Biodistribution in Non-human Primates Compared with Mice. Mol. Ther. *27*, 2018–2037.
9. Paşca, S.P. (2018). The rise of three-dimensional human brain cultures. Nature *553*, 437–445.
10. Lancaster, M.A., and Knoblich, J.A. (2014). Generation of cerebral organoids from human pluripotent stem cells. Nat. Protoc. *9*, 2329–2340.
11. Lancaster, M.A., Renner, M., Martin, C.A., Wenzel, D., Bicknell, L.S., Hurles, M.E., Homfray, T., Penninger, J.M., Jackson, A.P., and Knoblich, J.A. (2013). Cerebral organoids model human brain development and microcephaly. Nature *501*, 373–379.
12. Grenier, K., Kao, J., and Diamandis, P. (2020). Three-dimensional modeling of human neurodegeneration: brain organoids coming of age. Mol. Psychiatry *25*, 254–274.
13. Yoon, K.J., Song, G., Qian, X., Pan, J., Xu, D., Rho, H.S., Kim, N.S., Habela, C., Zheng, L., Jacob, F., et al. (2017). Zika-Virus-Encoded NS2A Disrupts Mammalian Cortical Neurogenesis by Degrading Adherens Junction Proteins. Cell Stem Cell *21*, 349–358.e6.
14. Zhang, B., He, Y., Xu, Y., Mo, F., Mi, T., Shen, Q.S., Li, C., Li, Y., Liu, J., Wu, Y., et al. (2018). Differential antiviral immunity to Japanese encephalitis virus in developing cortical organoids. Cell Death Dis. *9*, 719.
15. D'Aiuto, L., Bloom, D.C., Naciri, J.N., Smith, A., Edwards, T.G., McClain, L., Callio, J.A., Jessup, M., Wood, J., Chowdari, K., et al. (2019). Modeling Herpes Simplex Virus 1 Infections in Human Central Nervous System Neuronal Cells Using Two- and Three-Dimensional Cultures Derived from Induced Pluripotent Stem Cells. J. Virol. *93*, e00111-19.
16. Chen, H.I., Song, H., and Ming, G.L. (2019). Applications of Human Brain Organoids to Clinical Problems. Dev. Dyn. *248*, 53–64.
17. Watanabe, M., Buth, J.E., Vishlaghi, N., de la Torre-Ubieta, L., Taxidis, J., Khakh, B.S., Coppola, G., Pearson, C.A., Yamauchi, K., Gong, D., et al. (2017). Self-Organized Cerebral Organoids with Human-Specific Features Predict Effective Drugs to Combat Zika Virus Infection. Cell Rep. *21*, 517–532.
18. Garita-Hernandez, M., Routet, F., Guibbal, L., Khabou, H., Toualbi, L., Riancho, L., Reichman, S., Duebel, J., Sahel, J.A., Goureau, O., and Dalkara, D. (2020). AAV-mediated gene delivery to 3D retinal organoids derived from human induced pluripotent stem cells. Int. J. Mol. Sci. *21*, 994.
19. Kunze, C., Börner, K., Kienle, E., Orschmann, T., Rusha, E., Schneider, M., Radivojkov-Blagojevic, M., Drukker, M., Desbordes, S., Grimm, D., and Brack-Werner, R. (2018). Synthetic AAV/CRISPR vectors for blocking HIV-1 expression in persistently infected astrocytes. Glia *66*, 413–427.
20. Latour, Y.L., Yoon, R., Thomas, S.E., Grant, C., Li, C., Sena-Esteves, M., Allende, M.L., Proia, R.L., and Tifft, C.J. (2019). Human *GLB1* knockout cerebral organoids: A model system for testing AAV9-mediated *GLB1* gene therapy for reducing GM1 ganglioside storage in GM1 gangliosidosis. Mol. Genet. Metab. Rep. *21*, 100513.
21. Matos, C.A., de Almeida, L.P., and Nóbrega, C. (2019). Machado-Joseph disease/spinocerebellar ataxia type 3: lessons from disease pathogenesis and clues into therapy. J. Neurochem. *148*, 8–28.
22. Rossi, A., Dupaty, L., Aillot, L., Zhang, L., Gallien, C., Hallek, M., Odenthal, M., Adriouch, S., Salvetti, A., and Büning, H. (2019). Vector uncoating limits adeno-associated viral vector-mediated transduction of human dendritic cells and vector immunogenicity. Sci. Rep. *9*, 3631.
23. Pillay, S., and Carette, J.E. (2017). Host determinants of adeno-associated viral vector entry. Curr. Opin. Virol. *24*, 124–131.
24. Castle, M.J., Gershenson, Z.T., Giles, A.R., Holzbaur, E.L.F., and Wolfe, J.H. (2014). Adeno-associated virus serotypes 1, 8, and 9 share conserved mechanisms for anterograde and retrograde axonal transport. Hum. Gene Ther. *25*, 705–720.
25. Emborg, M.E., Hurley, S.A., Joers, V., Tromp, D.P.M., Swanson, C.R., Ohshima-Hosoyama, S., Bondarenko, V., Cummisford, K., Sonnemans, M., Hermening, S., et al. (2014). Titer and Product Affect the Distribution of Gene Expression After Intraputaminal Convection-Enhanced Delivery. Stereotact. Funct. Neurosurg. *92*, 182–194.
26. Green, F., Samaranc, L., Zhang, H.S., Manning-Bog, A., Meyer, K., Forsayeth, J., et al. (2016). Axonal transport of AAV9 in nonhuman primate brain. Gene Ther. *23*, 520–526.
27. Tardieu, M., Zérah, M., Gougeon, M.L., Ausseil, J., de Bournonville, S., Husson, B., Zafeiriou, D., Parenti, G., Bourget, P., Poirier, B., et al. (2017). Intracerebral gene therapy in children with mucopolysaccharidosis type IIIB syndrome: an uncontrolled phase 1/2 clinical trial. Lancet Neurol. *16*, 712–720.
28. Al-Zaidy, S., Pickard, A.S., Kotha, K., Alfano, L.N., Lowes, L., Paul, G., Church, K., Lehman, K., Sproule, D.M., Dabbous, O., et al. (2019). Health outcomes in spinal muscular atrophy type 1 following AVXS-101 gene replacement therapy. Pediatr. Pulmonol. *54*, 179–185.
29. Hudry, E., and Vandenberghe, L.H. (2019). Therapeutic AAV Gene Transfer to the Nervous System: A Clinical Reality. Neuron *101*, 839–862.
30. Gilkes, J.A., Bloom, M.D., and Heldermon, C.D. (2015). Preferred transduction with AAV8 and AAV9 via thalamic administration in the MPS IIIB model: A comparison of four rAAV serotypes. Mol. Genet. Metab. Rep. *6*, 48–54.
31. Aschauer, D.F., Kreuz, S., and Rumpel, S. (2013). Analysis of transduction efficiency, tropism and axonal transport of AAV serotypes 1, 2, 5, 6, 8 and 9 in the mouse brain. PLoS ONE *8*, e76310.
32. He, T., Itano, M.S., Earley, L.F., Hall, N.E., Riddick, N., Samulski, R.J., and Li, C. (2019). The Influence of Murine Genetic Background in Adeno-Associated Virus Transduction of the Mouse Brain. Hum. Gene Ther. Clin. Dev. *30*, 169–181.
33. Klaw, M.C., Xu, C., and Tom, V.J. (2013). Intraspinal aav injections immediately rostral to a thoracic spinal cord injury site efficiently transduces neurons in spinal cord and brain. Mol. Ther. Nucleic Acids *2*, e108.
34. Saraiva, J., Nobre, R.J., and Pereira de Almeida, L. (2016). Gene therapy for the CNS using AAVs: The impact of systemic delivery by AAV9. J. Control. Release *241*, 94–109.
35. Martier, R., Sogorb-Gonzalez, M., Stricker-Shaver, J., Hübener-Schmid, J., Keskin, S., Klima, J., Toonen, L.J., Juhas, S., Juhasova, J., Ellederova, Z., et al. (2019). Development of an AAV-Based MicroRNA Gene Therapy to Treat Machado-Joseph Disease. Mol. Ther. Methods Clin. Dev. *15*, 343–358.

36. Nguyen, H.P., Hübener, J., Weber, J.J., Grueninger, S., Riess, O., and Weiss, A. (2013). Cerebellar soluble mutant ataxin-3 level decreases during disease progression in Spinocerebellar Ataxia Type 3 mice. PLoS ONE *8*, e62043.

37. Watanabe, M., Buth, J.E., Vishlaghi, N., de la Torre-Ubieta, L., Taxidis, J., Khakh, B., Coppola, G., Pearson, C.A., Yamauchi, K., Gong, D., et al. (2017). Self-organized cerebral organoids with human specific features predict effective drugs to combat Zika virus infection. Cell Rep. *21*, 517–532.

38. Dekkers, J.F., Wiegerinck, C.L., de Jonge, H.R., de Jong, N.W.M., Bijvelds, M.J.C., Nieuwenhuis, E.E.S., et al. (2013). A functional CFTR assay using primary cystic fibrosis intestinal organoids. Nat. Med. *19*, 939–945.

39. Ormel, P.R., Vieira de Sá, R., van Bodegraven, E.J., Karst, H., Harschnitz, O., Sneeboer, M.A.M., Johansen, L.E., van Dijk, R.E., Scheefhals, N., Berdenis van Berlekom, A., et al. (2018). Microglia innately develop within cerebral organoids. Nat. Commun. *9*, 4167.

40. Lancaster, M.A. (2018). Brain organoids get vascularized. Nat. Biotechnol. *36*, 407–408.

41. Keskin, S., Brouwers, C.C., Sogorb-Gonzalez, M., Martier, R., Depla, J.A., Vallès, A., van Deventer, S.J., Konstantinova, P., and Evers, M.M. (2019). AAV5-miHTT Lowers Huntingtin mRNA and Protein without Off-Target Effects in Patient-Derived Neuronal Cultures and Astrocytes. Mol. Ther. Methods Clin. Dev. *15*, 275–284.

42. Miniarikova, J., Zanella, I., Huseinovic, A., van der Zon, T., Hanemaaijer, E., Martier, R., Koornneef, A., Southwell, A.L., Hayden, M.R., van Deventer, S.J., et al. (2016). Design, Characterization, and Lead Selection of Therapeutic miRNAs Targeting Huntingtin for Development of Gene Therapy for Huntington's Disease. Mol. Ther. Nucleic Acids *5*, e297.

43. Chen, C., Ridzon, D.A., Broomer, A.J., Zhou, Z., Lee, D.H., Nguyen, J.T., Barbisin, M., Xu, N.L., Mahuvakar, V.R., Andersen, M.R., et al. (2005). Real-time quantification of microRNAs by stem-loop RT-PCR. Nucleic Acids Res. *33*, e179.

OMTM, Volume *18*

Supplemental Information

Cerebral Organoids: A Human Model for AAV Capsid Selection and Therapeutic Transgene Efficacy in the Brain

Josse A. Depla, Marina Sogorb-Gonzalez, Lance A. Mulder, Vivi M. Heine, Pavlina Konstantinova, Sander J. van Deventer, Katja C. Wolthers, Dasja Pajkrt, Adithya Sridhar, and Melvin M. Evers

Supplemental figures

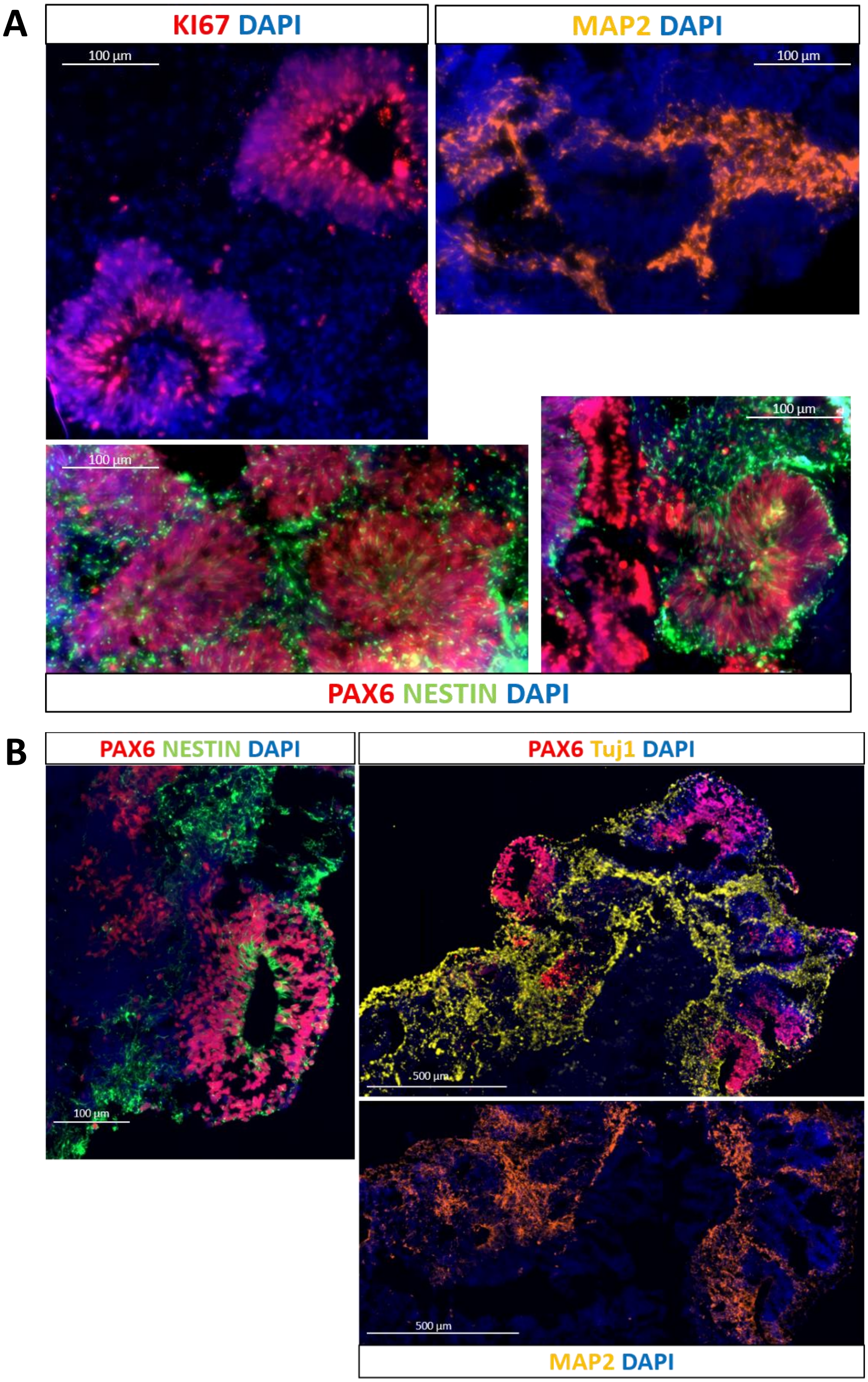

Figure S1: Generation of cerebral organoids. Images of immunostained sections of two 30 day old organoids from two different batches (A & B), show the presence of neural progenitors (PAX6), neurons (Tuj1 and MAP2) and proliferating cells (Ki67). Nuclei were dyed with DAPI.

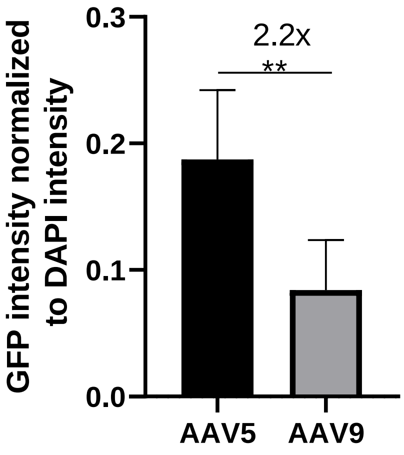

*Figure S2: Comparing rAAV5 and rAAV9 transduction efficiency in cerebral organoids. rAAV5 transduced organoids express GFP at a 2.2 times higher intensity compared to rAAV9 transduced organoids (**p<0.005). Total GFP intensity was measured in z-stacks of 12 regions per organoid. The intensity was normalized to the DAPI signal.*
